# Orthopædics "Napoo" at Leicester

**Published:** 1918-06-29

**Authors:** 


### ORTHOPÆDICS "NAPOO" AT LEICESTER.

In the House of Commons there lately appeared on the question paper an inquiry by Sir Maurice Levy, M.P., as to whether in response to an appeal by the Ministry of Pensions the Mayor of Leicester and the Board of Governors of the Royal Infirmary had consented to provide a sum of £20,000 for an in-patients' and an out-patients' orthopædic hospital for discharged soldiers, whether the offer had been declined by the Priority Committee of the Ministry of Munitions, and whether the Government was aware that there was no provision for the treatment of orthopædic patients in the East Midlands. The inquiry elicited an answer from the Pensions Minister, Mr. Hodge, that there was no institution of the kind in the East Midlands, that the Mayor of Leicester and the Board of Governors of the Royal Infirmary at Leicester were prepared to provide one, and that he regretted that the scheme, involving the construction of permanent buildings, could not, owing to the shortage of labour and materials, be allowed to proceed at the present time. A communication from the Ministry of Pensions gives the substance of the above answer, but indicates that the Priority Committee would be prepared to give a favourable decision to a temporary scheme.

### What the Local Press Says.

A report of the conference with the representative of the Ministry of Pensions and the Mayor and Governors of the Royal Infirmary has already appeared in The Hospital (April 27, page 69) with reference to the establishment of an orthopædic centre, and in view of the definite and ready response to the Ministry's statement, it can quite well be understood that the decision of the Priority Committee has aroused considerable interest and discussion at Leicester. The *Daily Post* states that if it were a question of erecting a picture-house or a private mansion the action of the Priority Committee would be perfectly proper, but this was a matter of preventing men who had fought for their country from becoming permanently crippled, to guard against which prompt treatment was everything. The lack of co-ordination and interworking between Government Departments was almost as bad now as in the early days of the war. A "day" had to be asked to ventilate the inadequacy of present allowances to soldiers' wives; apparently another will be required if an arbitrary decision is not to deprive disabled fighting-men of the treatment they require to recover the use of their limbs. The *Leicester Mercury* points out that an institution necessary in the opinion of one Department is held up by the decision of another Department, and while there is said to be no labour or materials at present for a hospital for crippled soldiers, in Bucks corn crops are rooted up and a hospital pulled down to make room for workshops which might have been erected elsewhere. This surely was one of the things that "no fellah can understand," especially when they were further gravely informed that, while money might be spent and materials used in adapting an old building for the purpose, neither could be provided for a new one. The *Leicester Mail* comments: Supposing there is no old building suitable and available which can be converted, are men suffering from orthopædic conditions sustained in their country's service to be allowed to become permanent cripples by the Government's rigid adherence to a decision, to save a little labour and money? It was to be hoped that the Mayor and the Governors of the Royal Infirmary would further press their claim.

The decision of the Priority Committee will, of course, receive in due course the consideration of the Mayor and the Board of Governors of the infirmary, and The Hospital will await with interest their statement on the position. It certainly seems incomprehensible that a generous offer to provide for the erection and equipment of an up-to-date department in a hospital which has already done much in setting aside accommodation for wounded and discharged soldiers should be declined, especially when, as in this case, it was made in direct response to an application by a Government Department. Moreover, the columns of The Hospital show that the infirmary has no unused buildings which can be adapted, and that the governors have already expended several thousand pounds on the purchase of adjoining property to provide a site for the projected Department.

